# Dopamine and its receptors play a role in the modulation of CCR5 expression in innate immune cells following exposure to Methamphetamine: Implications to HIV infection

**DOI:** 10.1371/journal.pone.0199861

**Published:** 2018-06-26

**Authors:** Liana Basova, Julia A. Najera, Nikki Bortell, Di Wang, Rosita Moya, Alexander Lindsey, Svetlana Semenova, Ronald J. Ellis, Maria Cecilia Garibaldi Marcondes

**Affiliations:** 1 San Diego Biomedical Research Institute, San Diego, CA, United States of America; 2 Department of Neurosciences, The Scripps Research Institute, La Jolla, CA, United States of America; 3 University of California San Diego, Department of Psychiatry, San Diego, CA, United States of America; University of Texas Rio Grande Valley, UNITED STATES

## Abstract

The Human Immunodeficiency Virus (HIV) infects cells in the Central Nervous System (CNS), where the access of antiretrovirals and antibodies that can kill the virus may be challenging. As a result of the early HIV entry in the brain, infected individuals develop inflammation and neurological deficits at various levels, which are aggravated by drugs of abuse. In the non-human primate model of HIV, we have previously shown that drugs of abuse such as Methamphetamine (Meth) increase brain viral load in correlation with a higher number of CCR5-expressing myeloid cells. CCR5 is a chemokine receptor that may be involved in increasing inflammation, but also, it is a co-receptor for viral entry into target cells. CCR5-expressing myeloid cells are the main targets of HIV in the CNS. Thus, the identification of factors and mechanisms that impact the expression of CCR5 in the brain is critical, as changes in CCR5 levels may affect the infection in the brain. Using a well-characterized in vitro system, with the THP1 human macrophage cell line, we have investigated the hypothesis that the expression of CCR5 is acutely affected by Meth, and examined pathways by which this effect could happen. We found that Meth plays a direct role by regulating the abundance and nuclear translocation of transcription factors with binding sites in the CCR5 promoter. However, we found that the main factor that modifies the CCR5 gene promoter at the epigenetic level towards transcription is Dopamine (DA), a neurotransmitter that is produced primarily in brain regions that are rich in dopaminergic neurons. In THP1 cells, the effect of DA on innate immune CCR5 transcription was mediated by DA receptors (DRDs), mainly DRD4. We also identified a role for DRD1 in suppressing CCR5 expression in this myeloid cell system, with potential implications for therapy. The effect of DA on innate immune CCR5 expression was also detectable on the cell surface during acute time-points, using low doses. In addition, HIV Tat acted by enhancing the surface expression of CCR5, in spite of its poor effect on transcription. Overall, our data suggests that the exposure of myeloid cells to Meth in the context of presence of HIV peptides such as Tat, may affect the number of HIV targets by modulating CCR5 expression, through a combination of DA-dependent and–independent mechanisms. Other drugs that increase DA may affect similar mechanisms. The implications of these epigenetic and translational mechanisms in enhancing HIV infection in the brain and elsewhere are demonstrated.

## Introduction

Both the Human Immunodeficiency Virus (HIV) and the Simian Immunodeficiency Virus (SIV) cross the blood brain barrier early after infection carried by macrophages, and infect Chemokine Receptor 5 (CCR5) -expressing myeloid cells such as microglia, in the central nervous system (CNS) [1]. Once in the brain, HIV genetic variants evolve to become distinct from the periphery [2], in part as a result of selective pressures from a distinctive CD8+ T cell repertoire [3], and from the local selection based on CCR5 tropism [4]. Such compartmentalization has been associated with neurological disorders in infected individuals [5, 6]. In addition, due to the fact that microglia are long-lived cells, the CNS becomes a source of low-rate, steady HIV replication, and viral persistence [4], while the penetration or diffusion of most anti-retroviral drugs (ARV) is either sub-optimal or carries neurological side effects [7–10]. Together, these factors make the CNS a reservoir for HIV infection that may be difficult to reach with eradication strategies.

In the CNS, CCR5 is expressed by infiltrating macrophages, including those that carry the virus across the blood brain barrier, and microglia. Importantly, the expression of CCR5 in brain innate immune cells is enhanced in individuals that are at risk of becoming infected, such as in Methamphetamine (Meth) abusers, as shown by us in the SIV macaque model of neuroAIDS. We described that a chronic Meth regimen causes the upregulation of CCR5 in some subpopulations of innate immune cells in the brain [11], particularly microglia and a subset of macrophages that express the typical CD11b and CD14 surface markers. Despite of being induced to upregulate CCR5 and other activation markers, these cells still retain low levels of CD16, suggesting an intermediate inflammatory phenotype [11]. Importantly, the ability of Meth to increase CCR5 also correlates with a higher viral load in the brain of SIV-infected macaques [11, 12]. Additionally, HIV inoculation in the presence of dopamine (DA), a neurotransmitter that is critically increased by Meth in the brain, led to infection of a larger proportion of macrophages, compared to virus alone [13]. In humans, a significantly increased ratio between HIV RNA and DNA levels has been reported in the brain of HIV-infected subjects with a history of Meth abuse [14]. This supports in vitro data showing increased viral transcription in Meth users [15, 16]. We hypothesize that Meth-stimulated dopamine release might be a key factor in the Meth-increased viral replication, by interfering with CCR5 expression levels.

Meth is a highly addictive illicit drug that, among other effects, increases the levels of dopamine (DA) in selected areas of the brain [17]. DA seems to play a role in also increasing the number of infiltrating macrophages in the brain [18, 19], which, in infected individuals, may increase the odds of a CNS infection. Importantly, innate immune cells are sensitive and responsive to this mediator, as they express DA receptors, and other molecules of the DA system [20]. While DA has been shown to facilitate the CCR5-mediated entry of HIV into innate immune cells [13, 21], its ability to upregulate CCR5, to even further increase the number of virus targets has not been examined. The promoter of the CCR5 gene has binding sites to transcription factors that are responsive to DA, but also to reactive oxygen species (ROS). Therefore, the effects of Meth on CCR5 expression could be mediated by either dopamine or ROS, particularly in the context of HIV.

Here we used an in vitro DA-free system of THP1 human macrophages to examine the hypothesis that Meth alone, or together with the highly active HIV-1 peptide Tat, up-regulates CCR5 directly through ROS, or indirectly upon the exposure to dopamine. First, we confirmed that ROS is produced acutely in our system, and that dopamine is not present in the cultures. Then we examined the ability of the drug to induce CCR5 transcription through ROS or through exogenous dopamine.

We found that transcription factors that are active in the CCR5 promoter are highly enhanced and mobilized both by ROS, which is acutely induced by Meth in innate immune cells, as well as by DA. Several reports have previously suggested that HIV Tat peptide has an effect in enhancing CCR5 expression. In our system HIV Tat was poor at inducing epigenetic changes, mobilizing transcription factors active at the CCR5 promoter, or increasing CCR5 transcription. But it did play a role in increasing surface levels of CCR5. It also decreased an isoform of DA receptor that showed a suppressor effect on CCR5 transcription, suggesting an indirect positive effect. On the other side, we found that DA is a key factor necessary to induce histone modifications that are compatible with a CCR5 enhanced transcription. Together, the results suggest that the combination of effects independently triggered by Meth, HIV Tat and DA can potentiate CCR5 expression, with a potential impact in the infection of innate immune cells, through increasing the number of CCR5+ target cells.

## Materials and methods

### Cell cultures

Human THP1 cells[22] were obtained from the American Type Culture Collection (ATCC), and were maintained in our lab, using RPMI-1640 medium (Gibco) supplemented with 10% fetal bovine serum (Hyclone), 2mM Glutamine (Gibco), 100U/ml penicillin/streptomycin (Gibco), and 0.05mM beta-2-mercaptoethanol (Sigma-Aldrich). Co-cultures were performed by incubating THP1 cells onto 2x10^5^ confluent SH-SY5Y human neurons, in 12 well plates (Corning). For that, the neurons were allowed to become confluent prior to the addition of THP1 cells. In some experiments, pre-exposure of the neurons to Meth was performed 2 hours before the addition of THP1 cells.

### Meth stimulation

Stimulation with (+)-methamphetamine hydrochloride (Sigma-Aldrich) was optimized and performed by adding 60uM of the drug in PBS to cultures containing 10^6^ cells per ml. The effects of the drug were examined on cells that were harvested at different time points following stimulation, from 15 minutes to 24 hours.

### Tat, NAC and DA treatments

Recombinant HIV Tat (clade B) was obtained from NIH AIDS Reagent Program. Tat was added at 10ng/ml to cells for the time indicated. N-acetyl cysteine (Sigma-Aldrich) was used in cultures at 5uM, 30 minutes prior to the addition of Meth. Dopamine Hydrochloride (DA, Sigma-Aldrich) was incubated for the indicated periods at the concentration of 1, 10 or 100uM. Optimal results were obtained with 1uM of DA.

### Determination of levels of catecholamine levels in the cultures

Dopamine, Epinephrine and Norepinephrine were measured in the supernatant of the cell cultures after Meth stimulation using liquid chromatography tandem mass-spectrometry (LC-MS/MS). Standard solutions were prepared with dopamine hydrochloride, (-)-epinephrine, or (-)-norepinephrine (all Sigma-Aldrich). Calibrator stock solutions and internal standards were prepared in hydrochloric acid 0.01 mol/l. The extraction in supernatants as well as in the culture media was improved by deproteinization with different solvents and two different pH values (8.5 and 9.5). The solvent and mixtures tested were ethyl acetate, chloroform:isopropyl alcohol (80:20), chloroform:diethyl ether (80:20) and chloroform:isopropyl alcohol (50:50). The sample volume used was changed for 1.0 ml and the elution volume for 250 μl of formic acid 1.0% for better sensibility. A standard solution with concentrations epinephrine 23.0 ng/ml, norepinephrine 90.0 ng/ml, and dopamine 415.0 ng/ml, prepared in solution of hydrochloric acid 0.01 mol/l were analyzed in triplicate for each solvent and solid phase cartridges. Standard curves of each catecholamine were acquired for linear regression. The pH was adjusted to 9.5 with ammonium hydroxide 5% and they were submitted to a vigorous stirring for 60 s using a vortex apparatus. 1500 μl of ethyl acetate was added in this solution and vortexed for more 60 s. This mixture was centrifuged (5 min at 2000 g) and 800 μl of the supernatant was evaporated with a vacuum concentrator. The extract was reconstituted with 200 μl of the mobile phase. Chromatography was performed on a Waters Alliance HT (Waters, Milford, MA) equipped with a BDS HYPERSILTM C18 column (125 mm × 3 mm, 3 μm particle size, Thermo Scientific). The isocratic mobile phase consisting of water:methanol (98:2 v/v) with 0.25% of formic acid pumped at a flow rate of 200 μl/min. The column was maintained at 30°C with a column oven. The injection volume was 20 μl. The method has a chromatographic running time of approximately 10 min. Detection was performed on a Quattro Micro tandem mass spectrometer triple quadrupole using a positive electrospray ionization (Waters, Milford, MA). To tune the mass spectrometer, a separately solution of epinephrine, norepinephrine, dopamine, and their respective internal standards in methanol:water (50:50, v/v) were infused directly into the mass spectrometer at a flow rate of 10.0 μl/min. The parameters were optimized to maximize detection.

### Protein extracts and western blots

Following a wash with ice-cold PBS, protein from cell cultures was extracted by lysis in radio-immunoprecipitation assay buffer (RIPA—Thermo Fisher Scientific, Waltham, MA) in the presence of Complete protease inhibitor cocktail tablets (Roche Molecular Biochemicals, Indianapolis, IN). The cells were scraped and transferred to a microfuge tube and spun at 10,000 rpm at 4°C for 10 min. The supernatant was transferred to a new tube and protein concentration was measured using a Bradford Reagent (BioRad, Hercules, CA). Protein was stored in −20°C until use. Ten micrograms of protein were loaded into each lane of SDS-PAGE electrophoresis gels (BioRad) in 4–20% gradient gels under reducing conditions. Transfer and immunodetection were performed as previously described [23]. Nonspecific antibody binding was blocked using 5% nonfat dried milk for 1 h at room temperature. Immunoblotting was carried out with antibodies against cJun (clone 60A8), p-cJun (cloneS63), Src (clone36D10), cFos (clone 9F6), p-cFos (cloneD82C12), NFkB p65 (clone D14E12), and IkB (cloneL35A5) (all Cell Signaling), p-Src (clone 1246F), and pEGFR/ErB1 (clone Y1068) (both from R&D Systems), pEGFR (clone 102618, Novus), as well as anti-beta actin (clone 13E5 (Cell Signaling), at concentrations suggested by manufacturers, followed by secondary antibody HRP-conjugated anti-rabbit IgG (Novus), or anti-mouse (Cell Signaling). Blots were developed in film (Kodak) with 1:1 solution of Super Signal West Pico Chemiluminescent Substrate and Luminol/Enhancer (Thermo Fisher Scientific, Rockford, IL). Bands were scanned and band intensities were calculated in ImageJ 1.43u (National Institute of Health, Bethesda, MD). Experimental bands were normalized to the intensity of beta-actin bands in each sample.

### Immunocytochemistry

The THP1 cells were brought to the concentration of 10^6^/ml and stimulated with 50 nM of phorbol-12-myristate-13-acetate for 24 hours for differentiation prior to the stimulation with Meth (60uM), DA (1uM) or HIV Tat (10ng/ml), on poly-L-lisine (Sigma Aldrich)-treated 8-well glass chamber slides (Thermo Scientific), and fixed with 4% paraformaldehyde for 20 minutes in the dark, ad then washed with PBS. Wells were then incubated with PBS containing 0.1% Triton X-100 for 15 minutes at room temperature, rinsed 3 times with PBS, and then blocked with 5g/l Casein (Sigma Aldrich) in PBS, containing 0.5g/l Thimerosal (Sigma Aldrich) for 1 hour at room temperature. The primary antibodies against transcription factors were the same as the ones used in western blots, and were diluted in blocking solution, and then placed in the wells for 2 hours at room temperature. Then cells were rinsed 3 times for 10 minutes with 1% blocking solution in PBS, followed by incubation with a secondary Alexa594-labeled donkey anti-rabbit or anti-mouse IgG (Thermo Fisher Scientific), for two hours at room temperature, in the dark. After rinsing, 4', 6-Diamidino-2-Phenylindole, Dihydrochloride (DAPI) was diluted to 300 ng/ml in 1% blocking solution for 10 minutes, in the dark. Cells were rinsed and maintained in PBS, and observed in a Nikon A1R laser-scanning confocal mounted onto a Nikon inverted Ti-E scope (Nikon, Melville, NY), and with a 20x PlanApo objective, 0.8NA (Nikon) and Images were acquired using a NIS-Elements C software (Nikon). Fluorescence intensity was normalized against background (secondary antibody only). Image analysis was performed in Fiji/ImageJ (National Institute of Health, USA). For that, tiff image files were opened and manually thresholded to identify stained cells. A binary mask was obtained from the negative thresholded DAPI image and applied to the total transcription factor stained area. The translocation index was calculated as percentage of the total transcription factor stained measurement values that is present within the nuclear area, and derived from the difference between total and nuclear staining.

### RNA Pol chromatin immunoprecipitation

Transcription rates were estimated as a function of RNA Polymerase II (RNAPII) occupancy combined with Next-Generation sequencing for a genome-wide readout, with a focus on the CCR5 gene. For that, the cells were harvested, washed in ice cold PBS and treated with1% formaldehyde for 12 min to crosslink the chromatin. The reaction was stopped with glycine added to a final concentration of 0.125 M. The pellets were lysed with lysis buffer (85 mM KCl, 0.5% NP40, 5 mM HEPES pH 8.0) supplemented with a protease inhibitor cocktail, incubated on ice for 15 min and centrifuged at 3500 *g* for 5 min to pellet the nuclei. The pellet was resuspended in nuclear lysis buffer (10 mM EDTA, 1% SDS, 50 mM Tris–HCl, pH 8.1) at a ratio 1:1 (v/w), incubated on ice for 10 min, and stored at −80°C until use for RNApol-ChIP. The chromatin extraction was performed using Chromatin IP DNA Purification kit (Active Motif, Carlsbad, CA). ChIP was performed using the ChIP-IT High Sensitivity Kit (Active Motif) with 30 μg of chromatin and 4 μg of antibody anti-RNA PolII Clone: 1F4B6 (Active Motif). ChIP Seq libraries were prepared and ChIP DNA was sequenced on the Illumina HiSeq using Active Motif Epigenetic Services. For the analysis, the data files were normalized to the same number of unique alignments without duplicate reads, which was 16 million sequence tags mapped to identify RNA pol II binding sites. Intervals (peaks, “islands”) were determined using the SICER algorithm at a cutoff of FDR1E-10 and a Gap parameter of 600 bp (which merges peaks located within 600 bp of each other into a single “island”). The number of intervals identified ranged from 9,775 in samples Meth+NAC to 14,124 in samples Meth+Tat. Data set was visualized in the UCSC Genome Browser.

### Histone modifications in the CCR5 promoter

ChIP-Seq was used to determine changes in histone modifications within the CCR5 promoter sequence. Cell crosslinking and chromatin extraction were performed as above. Antibodies were against H3K9ac, H3K4ac, H3K27ac and H3K27met (5 ul/ ChIP, All Active Motif). Following binding, real time, quantitative PCR (RT-qPCR) was performed on DNA purified from each of the ChIP reactions using a primer pair specific for the indicated gene regions. The CCR5 promoter sequence and annotation [24] (Entrez ID:1234) was utilized to design the primers to 500 bp apart segments, which span the entire promoter regions within the CCR5 gene sequence, and covering all 4 exons, as follows: 1) -1971 to -1852 bp: ctgtttaaagacaaaaaggccccaaaaaggagggatggcacgaaacaccctccaatatgggcatggagtctagagtgacaaagtgatcaaaagttcatttcctatggggtgtccgaatgt; 2) -1432 to -1313: aagcttgggcagtggaagtatcttgccgaggtcacacagcaagtcagcagcacagcgtgtgtgactccgagcctgctccgctagcccacattgccctctgggggtgagtatgtcttcaca; 3) -893 to -774: taatttggtcagagccaagtagcagtaatgaagctggaggttaaacccagcagcatgactgcagttcttaatcaatgccttttgaattgcacatatgggatgaactagaacattttctcg; 4) -353 to -235: gagggtaagacaggtttcaagcttggcagtctgactacagaggccactggcttagcccctgggttagtctgcctctgtaggattgggggcacgtaattttgctgtttggggtctcatttg; 5) 185 to 304: taaaactctttagacaacaggttttttccgtttacagagaacaataatattgggtggtgagcatctgtgtgggggttggggtgggataggggatacggggagagtggagaaaaagggggc; and 6) 724 to 843: taactccaccctccttcaaaagaaacagcatttcctacttttatactgtctatatgattgatttgcacagctcatctggccagaagagctgagacatccgttcccctacaagaaactctc. Data are presented as Fold Enrichment of the ChIP antibody signal versus the negative control IgG using the ddCT method.

### RNA extraction and qRT-PCR

Total RNA was isolated from the cells using RNAeasy Mini kit (Qiagen), according to the manufacturer’s instructions. Total RNA concentration was measured using the Nanodrop spectrophotometer and then used for reverse transcription using SuperScript III Reverse Transcriptase (Invitrogen, Waltham, MA). Most primers were purchased from Qiagen (Valencia, CA). PCRs were performed using RT2 SYBR Green ROX FAST Mastermix (Qiagen), in a 7900HT Fast Real-Time PCR System with Fast 96-Well Block Module (Applied Biosystems, Foster City, CA), with a SDS Plate utility v2.2 software (Applied Biosystems). The results were normalized to the expression of GAPDH. The primers for the DA receptors were previously described [18]. Primers for CCR5 were purchased from Qiagen.

### Production of virus HIV-pR7-GFP

The pR7-GFP HIV-1 was obtained from the National Institute of Health AIDS Reagent Program (# 3622). Max Efficiency Stbl2 Competent cells (Invitrogen) were used for cloning. The pR7-GFP DNA was isolated using NucleoBond Xtra Midi (Macherey-Nagel). The virus was then transfected into 293T cells maintained in DMEM with high glucose, supplemented with 10% Fetal Bovine Serum (Hyclone) and Penicillin/Streptomycin/Glutamine (Gibco), PEI (Polyethylenimine) Max (Polysciences, Inc). For that 293T cells were plated one day before transfection at 2 x 10^6^ cells in 75 cm^2^ flasks. On the day of the transfection, 2 ml of serum-free DMEM were mixed with pR7-GFP DNA and PEI Max, at a 1:2 DNA:PEI ratio, followed by a 20–30 min incubation at room temperature. The DNA/ PEI mixture was diluted to 11 ml with complete media, and added to 293T cells that were washed in 1x PBS. The cells were then incubated at 37oC for 2 hours. Following that, the DNA:PEI containing media were removed, and replaced with 25 ml of complete fresh media containing 1% Fetal Bovine Serum. The cells were then incubated for 48 hours. The supernatant was collected, spun at 1150 rpm for 5 min at RT to remove debris, and HIV-1 p24 was measured by HIV-p24 ELISA Assay (XpressBio).

### THP 1 infection, dopamine treatment and CCR5 dependence

THP 1 cells were washed with 1x PBS and placed into 48 well plate at 100 000 cells/400 μl. Fresh HIV-1 pR7-GFP virus was added at a concentration of 10 ng p24/ml. Dopamine at 1 μM, in the presence or absence of 0.1uM or 1uM of the CCR5 blocking compound TAK799 (Tocris) were added simultaneously with the virus. Infection was achieved using the spinoculation procedure (www.systembio.com), performed as described[25], for 2 hours at 2400 rpm at 32°C. Following the spinoculation, the THP 1 cells were resuspended in DMEM, and incubated for additional 48 h prior to the detection of GFP-labeled virus by Flow Cytometry.

### Flow cytometry

The surface expression of CCR5 was performed on cells that were incubated for 2, 6, 12 or 18 hrs with Meth (60uM), DA (1-100uM), Tat (10ng/ml), alone or in combination. The results displayed are from 2 hr incubations, when the effects were detectable and robust. For that, cultured cells were washed in staining buffer containing 2% fetal bovine serum and 0.2% sodium azide, and stained with an APC-labeled antibody against human CCR5 (CD195, clone 3A9) (BD Pharmingen, San Diego, CA), and with a PE-labeled anti-CD11b (clone M1/70) (Biolegend, San Diego, CA), as described[11]. The THP1 cells that were infected with HIV-1 pR7-GFP were washed 2 times with staining buffer. All the cells were fixed in 4% paraformaldehyde prior to acquisition in a BD LSR Fortessa X20 18-color FACS Analyzer, and the expression analysis, or calculation of infection rates, were performed in FlowJo software (Ashland, OR). CCR5 surface density was estimated by the ratio between geometric mean fluorescence and events counts within the viable forward versus side scatter gate.

### Statistical analysis

Group comparisons for individual genes across different conditions were performed using one-way or two-way ANOVA, followed by Bonferroni’s post hoc tests. The difference between the means was considered significant at p < 0.05. Tests were performed using Prism software (GraphPad Software, San Diego, CA, USA) for Macintosh.

## Results and discussion

THP1 human macrophages were used to model the effects of Meth on CCR5 transcription in the context of HIV infection. The model consisted of exposure of THP1 cells plated at a density of 2x10^6^/ml to 60uM Meth, a concentration that has been estimated to reach the brain in typical humans who use Meth, assuming a 3–15 mg/kg/day consumption[11], and an average of 0.04% of the injected dose reaching the brain within 7–10 minutes and slow clearance[26, 27].

**Fig 1:**
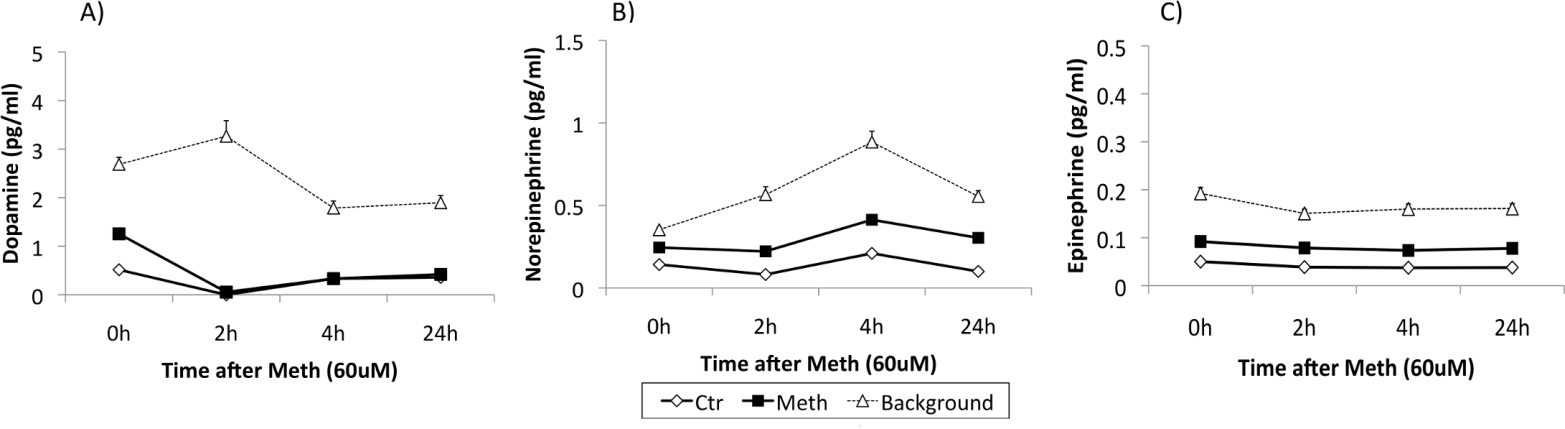
Meth does not induce catecholamines in innate immune cells in culture. **(A)** Dopamine, **(B)** Norepinephrine and **(C)** Epinephrine were measured in the supernatant of the cell cultures after Meth stimulation using liquid chromatography tandem mass-spectrometry (LC-MS/MS). The supernatant of control and Meth-stimulated THP1 cells was harvested at baseline, 2 hours, 4 and 24 hours, and the levels of catecholamines were plotted against background measurements obtained with culture media. The values of the supernatant of control or Meth-stimulated cells were below the detection threshold, suggesting that Meth does not induce catecholamine release from innate immune cells.

In vivo, acute Meth exposure induces neuronal production of dopamine (DA). DA can potentially influence the levels and activity of transcription factors, including the ones with binding sites in the CCR5 promoter. It has been reported that DA could potentially be produced by immune cells in a few conditions [28]. Thus, we examined whether Meth can induce DA in THP1 cell cultures (Fig 1A). Meth did not induce DA in our cultures, making this system ideal to distinguish potential DA-dependent and independent effects of Meth exposure on the expression of CCR5.

In addition to DA, we also examined whether Meth could induce other catecholamines in innate immune cells in culture. We measured levels of norepinephrine (Fig 1B) and epinephrine (Fig 1C) released in the supernatant of controls and Meth-treated cells. These catecholamines were not induced by Meth.

**Fig 2:**
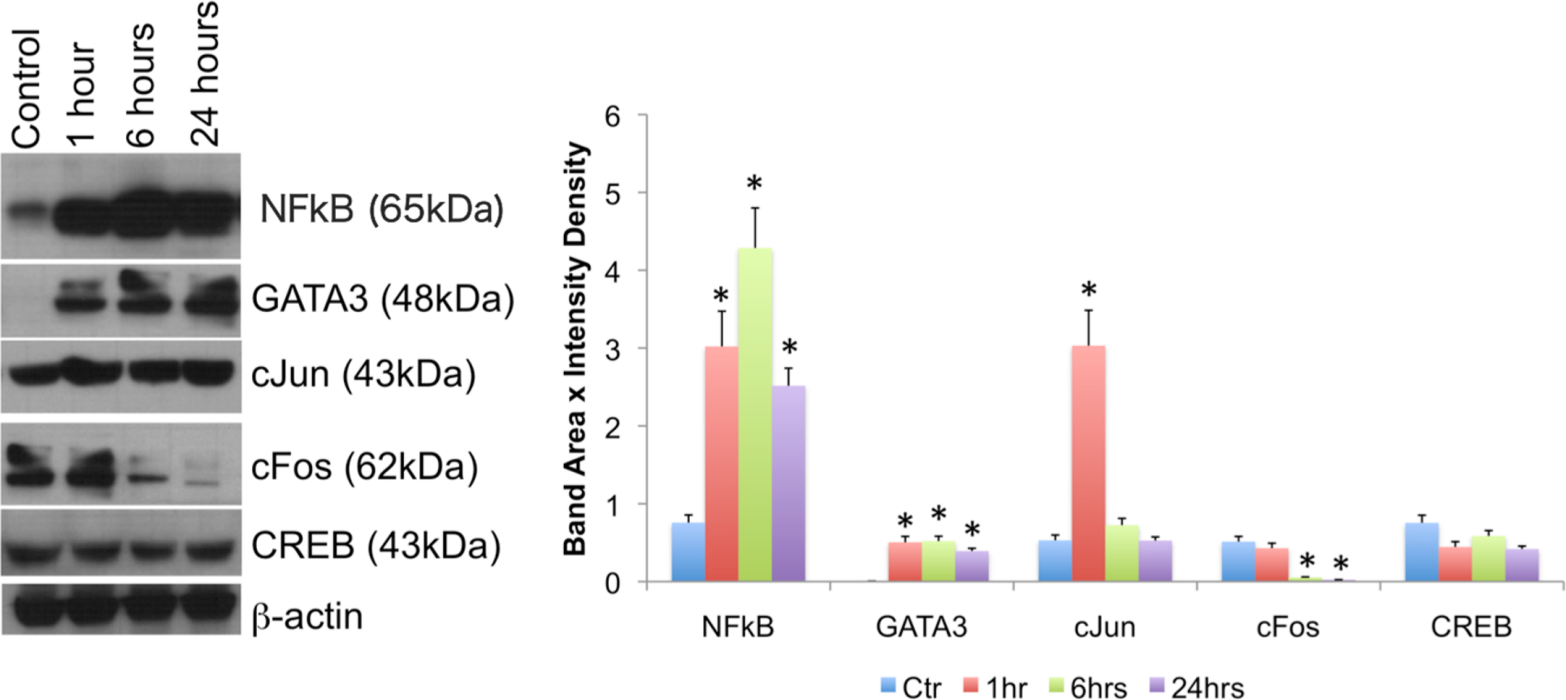
Meth affects levels of transcription factors with binding sites in the human CCR5 promoter. **(A)** Western blot was used to examine the enrichment of transcription factors such as NFkB, GATA3, cFos, and CREB, among others, normalized against b-actin, over time. The picture shows westerns performed in one representative experiment out of 3 independent tests. **(B)** Changes in the abundance of transcription factors were estimated by band area and intensity density. Values represent the average of at least three separated experiments. *p<0.05 compared to controls.

The stimulation of THP1 cells with Meth, and in the absence of catecholamines, affected the levels and translocation of transcription factors that have been described to be involved in CCR5 transcription, and that have mapped binding sites in the CCR5 promoter, suggesting a direct effect. Meth highly increased the levels of NFkB and GATA3, while decreasing cFos. There was strong effect of Meth on the levels of cJun at 1 hr, but no significant effect was observed on the levels of CREB, as examined by western blot (Fig 2).

**Fig 3:**
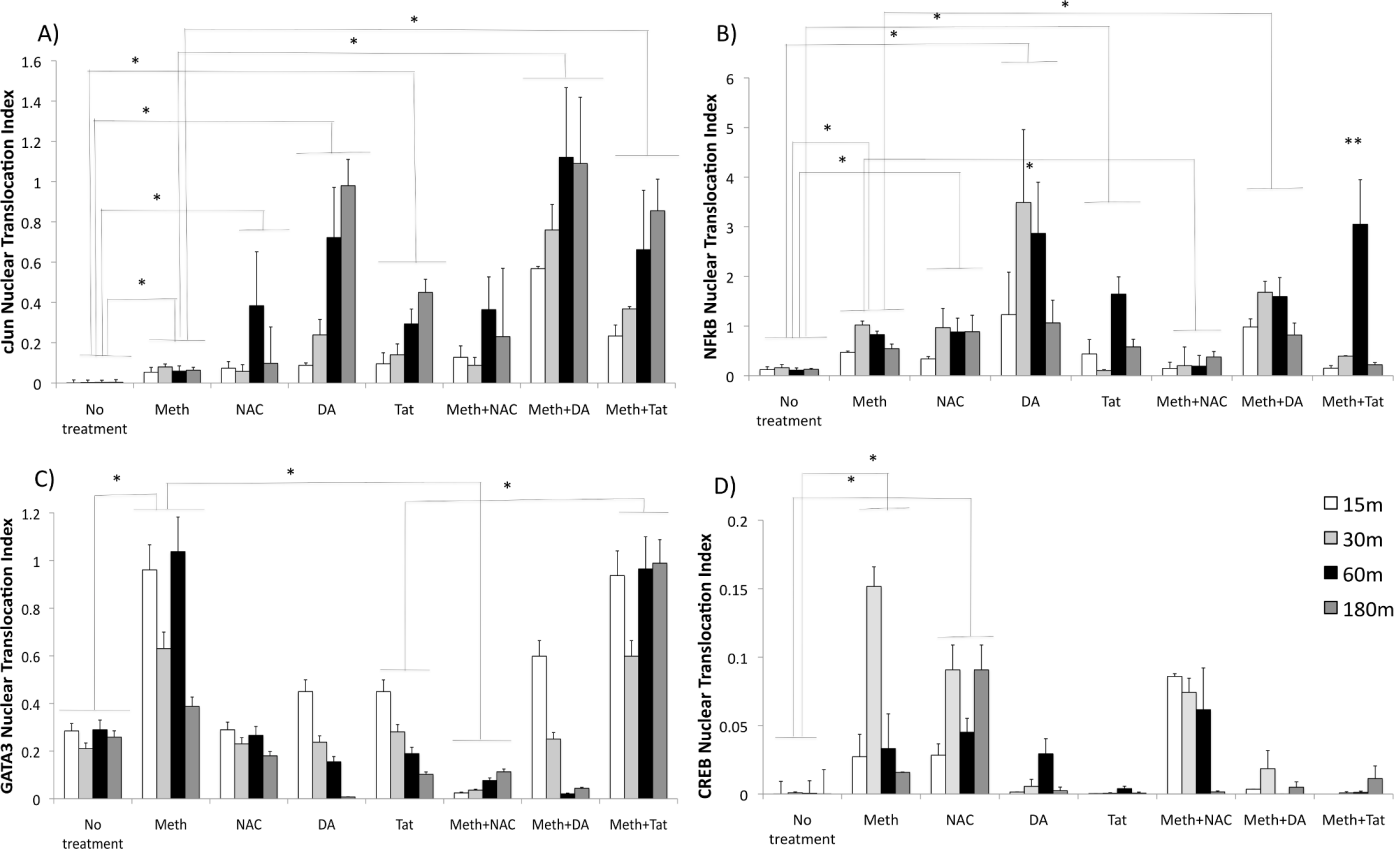
Nuclear translocation of CCR5-relevant transcription factors by Meth, dependent on ROS, effects of HIV Tat and of DA. Translocation of **(A)** cJun, **(B)** NFkB, **(C)** GATA3 and **(D)** CREB in THP1 macrophages treated with 60uM Meth, 5uM NAC, 10ng/ml HIV Tat or 1uM DA. Transcription factors were detected at different time points with immunocytochemistry, analyzed using ImageJ using confocal images. A nuclear translocation index was derived as percentage of the total transcription factor stained measurement values that is present within the nuclear area, and derived from the difference between total and nuclear staining (See S1 Fig for model). Nuclear translocation indexes from 5 experiments were compared using two-way ANOVA, followed by Bonferroni’s posthoc test.

The ability of Meth to induce the nuclear translocation of transcription factors was examined through immunocytochemistry and confocal imaging. The effects of Meth were compared with the effects of HIV Tat, as well as with the effects of exogenous DA, for mimicking factors present in the brain environment in the context of HIV infection. A translocation index was derived from the ratio between total transcription factor fluorescence intensity and nuclear fluorescence. Nuclear fluorescence intensity was subtracted from the total using ImageJ software, with the aid of a mask designed on images of DAPI nuclear staining. Using this approach, we examined native and phosphorylated forms of cJun and cfos, p38 MAPK, NFkB, GATA3 and CREB. We found that Meth could induce activation and translocation of all the transcription factors, even in the absence of DA. In addition, we found that DA and Tat translocate different sets of transcription factors, and with different intensity compared to Meth. For instance, DA and Tat were better than Meth at translocating cJun (Fig 3A) and DA was better in NFkB translocation (Fig 3B). On the other hand, Meth was better at translocating GATA3 (Fig 3C) and CREB (Fig 3D). The treatments did not significantly affect the traslocation of p38 MAPK or cFos (not shown).

Cells were treated with an antioxidant, NAC, to evaluate whether the nuclear translocation induced by Meth was dependent on the production of ROS in macrophages. Only NFkB (Fig 3B) and GATA3 (Fig 3C) were abolished by the anti-oxidant, suggesting that the translocation of these factors is associated with the production of ROS that is induced by Meth, in a DA-free environment. Interestingly, NAC alone or with Meth further increased the translocation of CREB (Fig 3D).

Tat combined with Meth potentiated the translocation of cJun at all time points (Fig 3A) and NFkB at 1 hr of stimulation, and prolonged GATA3 translocation (Fig 3C). DA together with Meth potentiated cJun (Fig 3A) and NFkB (Fig 3B) translocation.

**Fig 4:**
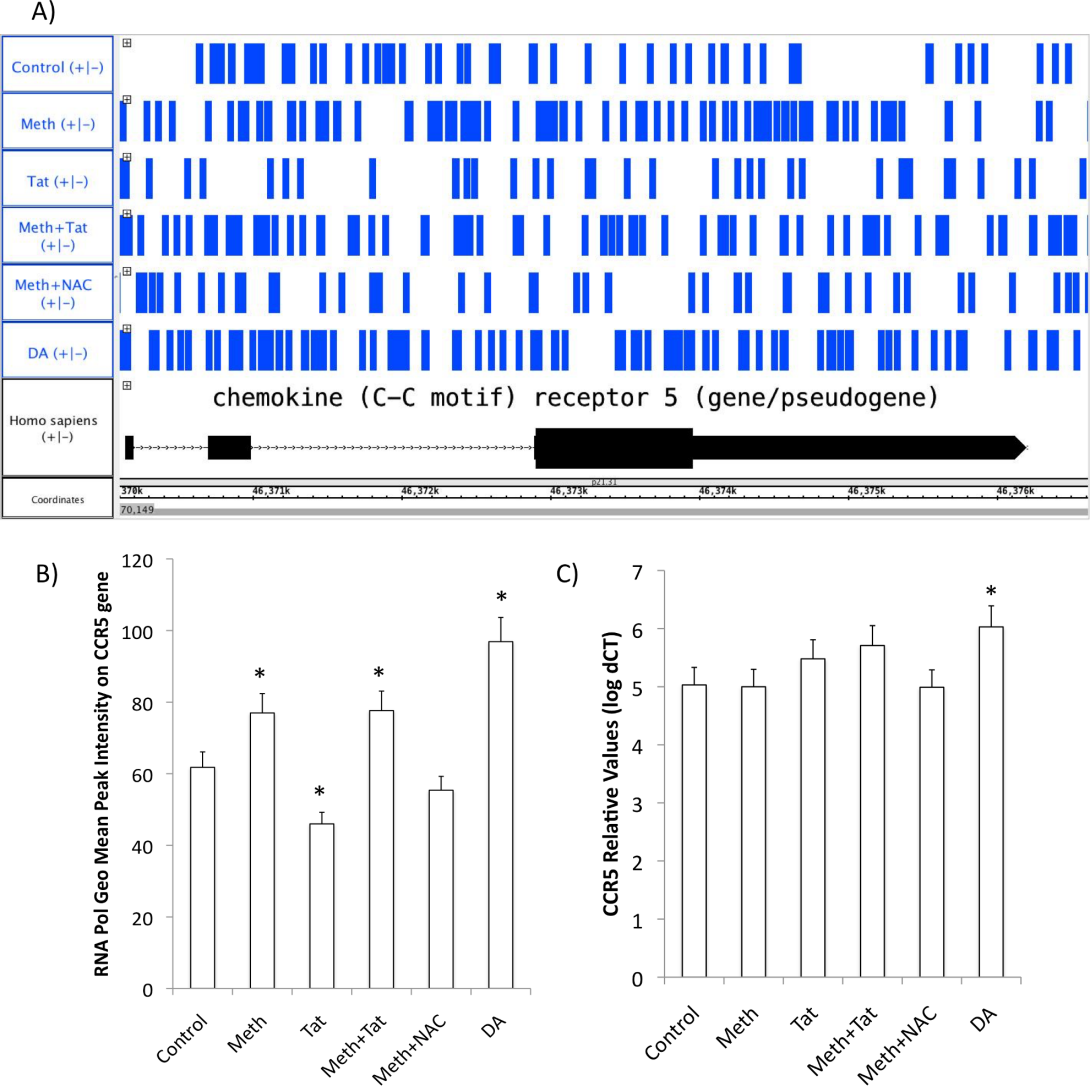
RNA Pol accumulation and transcription of the CCR5 gene. The pattern of RNA Pol II accumulation is a measure of the earliest detectable impact on the CCR5 promoter towards the gene transcription. **(A)** RNA Pol II accumulation pattern mapped onto the CCR5 gene sequence detected by RNA Pol ChIP-qPCR in THP1 cells stimulated with Meth (60uM), Tat (10ng/ml), or NAC (1nM), or DA (10nM) for 15 minutes before cell fixation and cross linking. Graph shows an Integrated Genome Browser (IGB 8.5) view of RNA Pol peak sequencing, and the alignment with the CCR5 sequence in the Homo sapiens genome. **(B)** Geometric mean of the total RNA Pol accumulation in the CCR5 gene in different conditions. **(C)** The effect of Meth, Tat and DA, on CCR5 transcription 2 hours after stimulation. CCR5 transcription was examined using SyBrGreen qRT-PCR, and normalized against the expression of housekeeping GAPDH. *p<0.05 compared to control conditions, One way ANOVA followed by Bonferroni’s posthoc test.

**Fig 5:**
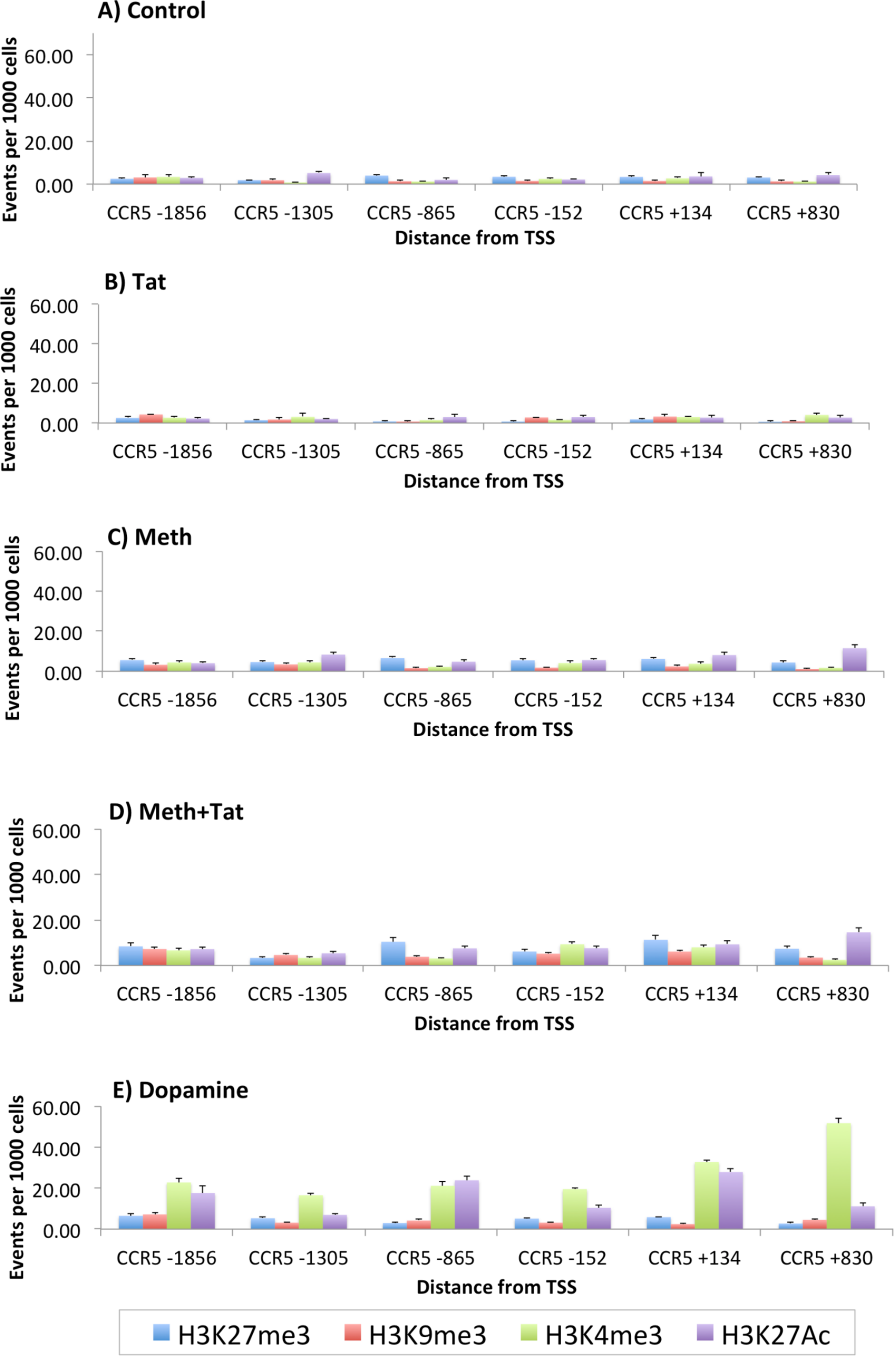
Histone modification patterns in CCR5 promoter regions, in THP1 cells treated with Meth, Tat and DA. Regulatory and enhancer histone modifications were detected by ChIP-qPCR, 30 minutes after stimulation. Suppressor H3K27me3 and H3K9me3, and enhancer H3K4me3 and H3K27Ac, were detected with specific antibodies on cross-linked chromatin, followed by qPCR to amplify 6 non-overlapping 500bp segments that covered the whole CCR5 gene. **(A)** Controls, **(B)** Meth, **(C)** Tat, **(D)** Meth+Tat, **(E)** DA.

Although Meth increased and mobilized relevant transcription factors, with and without Tat, CCR5 transcription was not significantly increased by these factors. This was confirmed by measuring the earliest transcriptional events, such as RNA polymerase accumulation on the CCR5 promoter transcription-starting site (TSS) 15 minutes after stimulation (Fig 4A and 4B), and by qRT-PCR at 2 hours after stimulation (Fig 5C). Interestingly, exogenous DA increased RNA Pol at higher levels compared to Meth or Meth+Tat (Fig 4A and 4B), and was able to significantly increase the CCR5 transcription (Fig 4C). This result suggests that even though Meth, with or without Tat, can mobilize transcription factors that are relevant to CCR5 transcription, and promote modest RNA Pol accumulation, this does not affect transcription. The following experiments investigated the hypothesis that missing signals that lead to CCR5 transcription can be provided by DA.

Epigenetic changes are key for allowing transcription factor binding, and can be estimated by the determination of histone modifications. Thus, we examined the CCR5 promoter for the induction of histone modifications that are compatible with regulated or enhanced transcription upon culture conditions of Meth, Tat or DA stimulation (Fig 5). The suppressor modifications examined were H3K27me3 and H3K9me3, and the enhancer modifications examined were H3K4me3 and H3K27Ac. Compared to Controls (Fig 5A), Tat stimulated cells (Fig 5B) did not present differences in histone modification patterns or levels, suggesting that Tat alone does not act on CCR5 transcription in our model. Meth stimulation caused a modest but significant increased of the number of cells with H3K27Ac, in regions +134 and +830 (p = 0.032), with or without Tat (Fig 5C and 5D). On the other hand, DA induced a robust increase in enhancing histone modifications in all promoter segments (p<0.0001). These data suggest that DA is a key factor in the increase of CCR5, by promoting epigenetic modifications that enable transcription factor binding and transcription.

**Fig 6:**
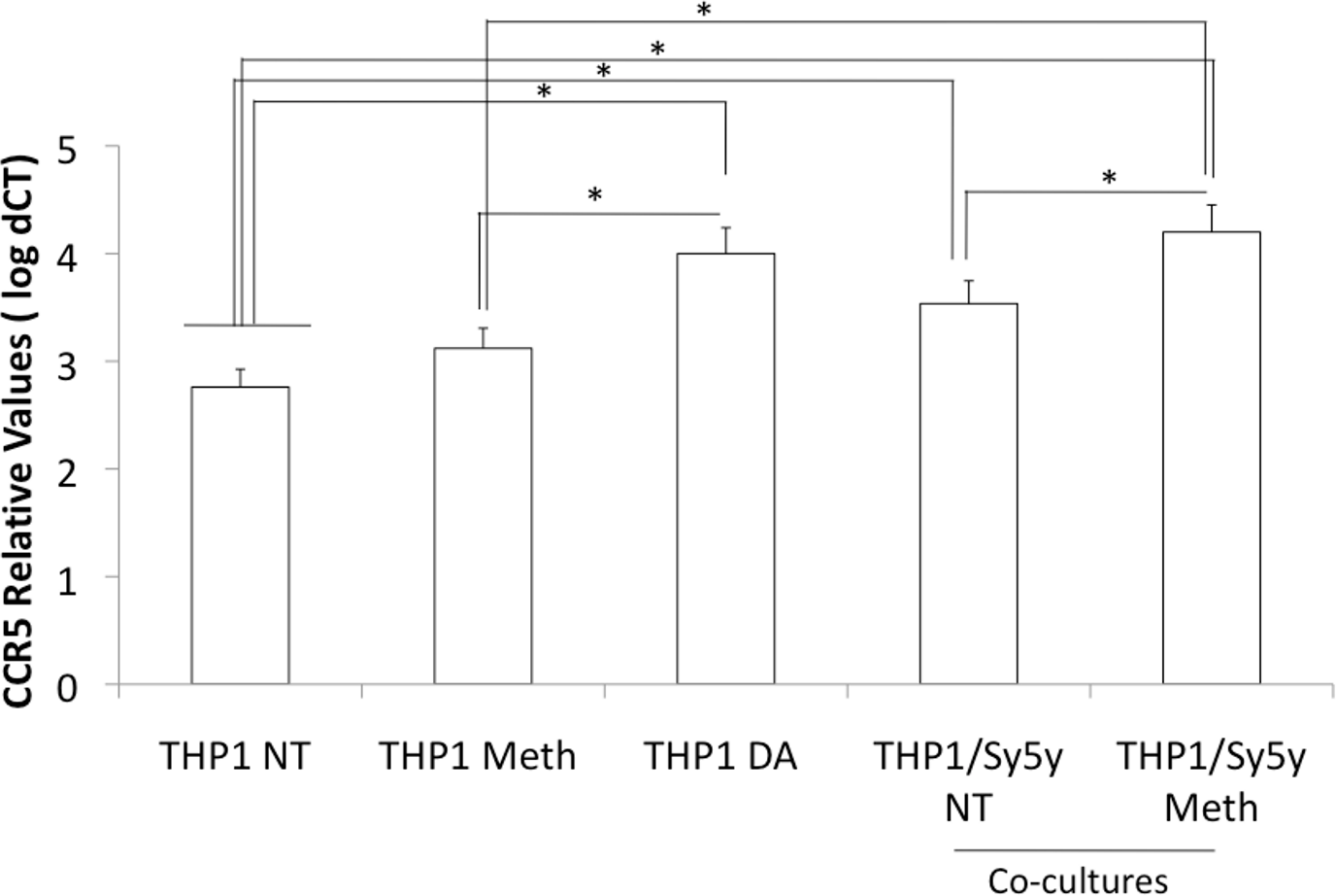
Effect of DA neurons and Meth stimulation on innate immune CCR5 transcription. CCR5 gene transcription was examined in THP1 macrophages, treated with Meth or DA (1 uM), or co-cultured with SH-Sy5Y DA neurons, with or without a 1 hour pre-stimulation with Meth (60uM). Results normalized to the expression of GAPDH, represent the average ± SD of 2 experiments performed in quadruplicate. *p<0.05 in Bonferroni’s post hoc test.

To further confirm that DA is a factor that could lead to CCR5 upregulation in vivo, we performed co-cultures of THP1 innate immune cells and a human dopaminergic neuronal cell line, SH-SY5Y (Fig 6). In co-cultures, the presence of dopaminergic neurons was able to increase CCR5 expression. This effect was enhanced in dopaminergic neurons that were pre-exposed to Meth for 1 hour prior to the addition of THP1 to co-cultures. This suggests that DA derived from neurons is able to increase CCR5 in innate immune cells. Such increases would be predicted to augment the number of susceptible host cells, potentially expanding the pool of cells harboring provirus.

**Fig 7:**
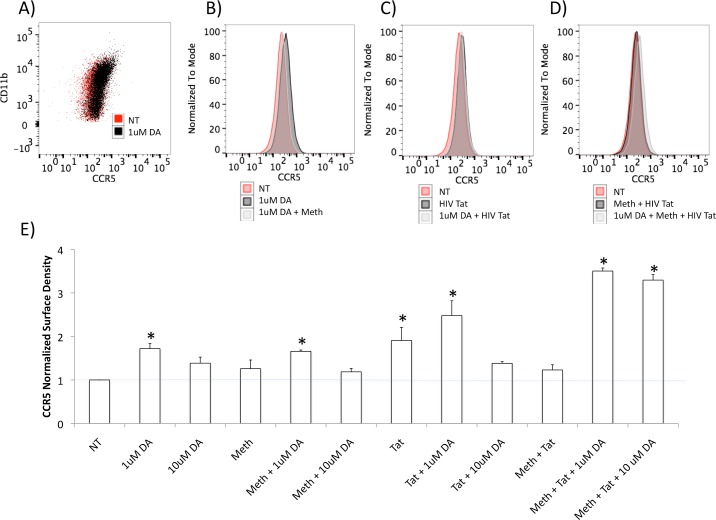
Modulation of the surface expression of CCR5 in THP1 cells by Meth, Tat and DA. FACS analysis was performed on THP1 cells stimulated with 1 or 10 uM of DA for 2 hrs, alone or in combination with Meth (60uM) and HIV Tat (10ng/ml). A) Representative scatter plot showing the expression of CCR5 and of the myeloid marker CD11b in cells that were not-treated (NT) or treated with 1uM DA. B) Overlapping representative histograms showing the CCR5 fluorescence intensity of NT, DA-treated cells (1uM) and DA+ Meth-treated cells. C) Overlapping representative histograms showing CCR5 fluorescence intensity in NT, Tat and DA (1uM) + Tat-treated cells. D) Overlapping representative histograms showing CCR5 fluorescence intensity in NT, and treatments with Meth + Tat, or Meth + Tat + DA (1uM) simultaneously in THP1 cells. *p<0.05 in comparison to NT. E) CCR5 density per cell was obtained by normalizing the geometric mean fluorescence to the number of acquired cell counts. Results represent the average ± SEM of 3 experiments performed in triplicate.

We then examined whether the upregulation of CCR5 induced by DA at the transcriptional level could be also observed on the cell surface. Using flow cytometry, we found that the increase in the surface expression of CCR5 is detectable after 2 hrs of exposure to the lower dose of DA tested (Fig 7). Higher doses of DA did not increase CCR5 on the surface, and in some experiments even caused a downregulation (not shown). The increase in CCR5 expression by 1uM of DA was no longer observed at 12 hrs after DA exposure (not shown), suggesting that this effect is limited to acute time points. Meth did not affect the surface expression of CCR5, as expected, alone or in combination with low doses of DA. HIV Tat, on the other hand, did increase its expression on the surface, in spite of the marginal effect on transcription. We calculated a function of the geometric mean fluorescence and the number of acquired cell counts to obtain an estimate of the CCR5 density per cell. With this calculation, we observed that when Tat was combined with DA, the density of CCR5 expression on the cell surface increased by 2.5 fold compared to non-treated controls (Fig 7E). When the THP1 cells were exposed to Meth, Tat and DA simultaneously, the CCR5 density increased above 3 fold, when compared to non-treated controls (Fig 7E). This suggests that there are effects of Tat on the expression of CCR5, and that Tat can further enhance the ability of DA to modulate CCR5, in spite of a poor effect on CCR5 transcription.

**Fig 8:**
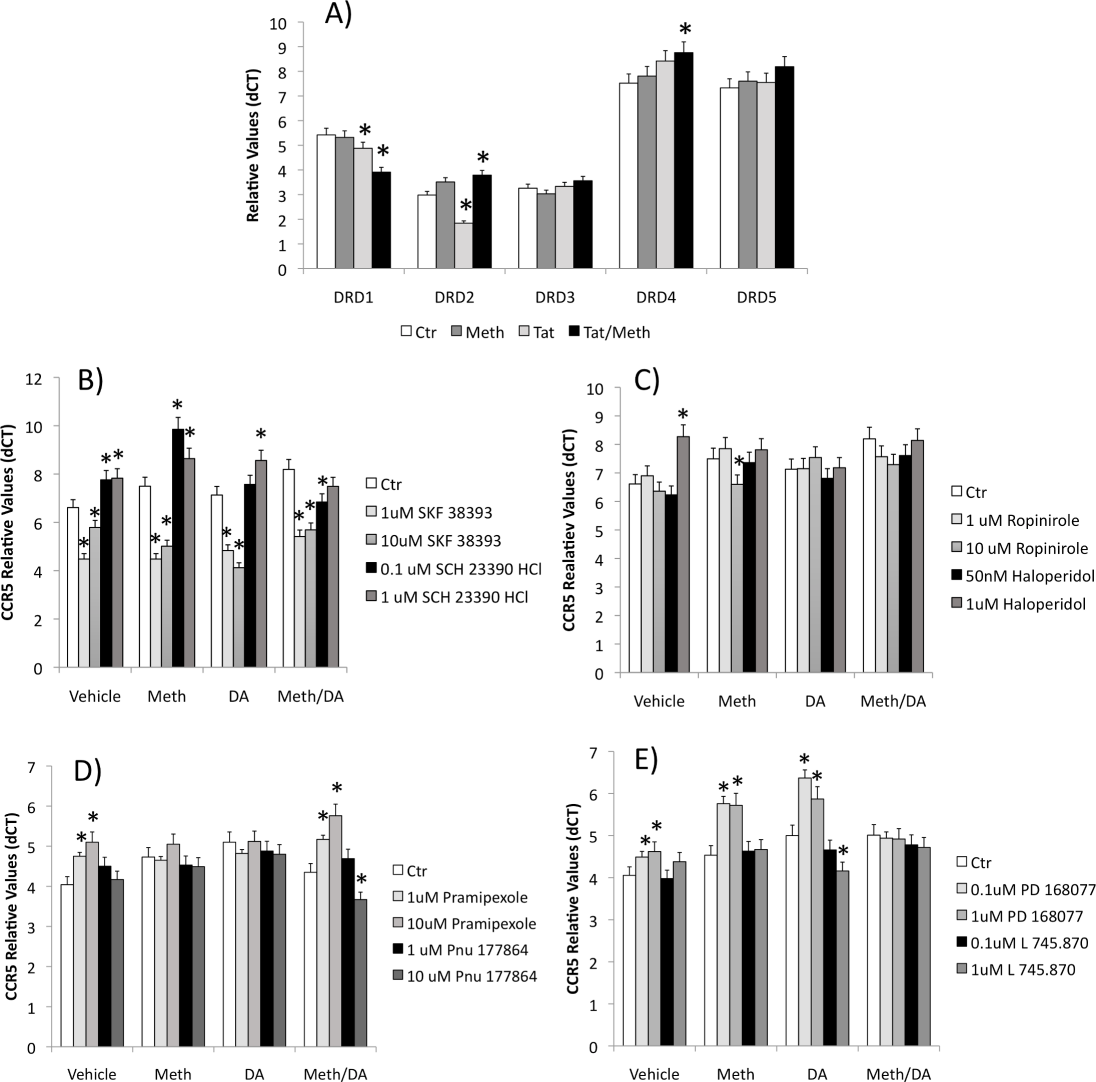
Expression of DRD receptors by THP1 cells and their role in the modulation of CCR5 transcription. **(A)** Transcription of all 5 DRDs on THP1 macrophages, in the presence or absence of Meth and/or HIV Tat peptide. THP1 cells were incubated with vehicle, Meth, DA or Meth and DA for 2 hrs, in the presence or absence of specific DRD receptor agonists and antagonists. Using qRT-PCR, CCR5 expression was tested upon a 30 min pre-treatment with **(B)** DRD1-like agonist SKF38393 or antagonist SCH 23390 HCl; or **(C)** DRD2 agonist Ropinirole or antagonist Haloperidol; or **(D)** DRD3 agonist Pramipexole or antagonist Pnu 177864; or **(E)** DRD4 agonist PD168077 or antagonist L745,870. Results represent the average ± SD of 3 experiments performed in duplicate. *p<0.05 compared to Control (Ctr) conditions within each treatment, ANOVA followed by Bonferroni’s test.

Using pharmacological tools, agonists and antagonists of DA receptor (DRD) isoforms, we dissected the relative contribution of individual DRDs to the increase in CCR5. We confirmed that THP1 cells express all the DRDs described in humans, DRD1 –DRD5 [18] at detectable levels, although the relative amounts differed from primary human monocytes (not shown). Furthermore, we found that the expression of DRD1 and DRD2, can be modulated by the exposure of cells to HIV Tat alone or together with Meth, while DRD4 is increased by HIV Tat and Meth simultaneous exposure (Fig 8A).

The ability of DRDs to signal CCR5 transcription in the context of Meth was tested in cells exposed to DA in the presence of specific DRD1-like (DRD1 and DRD5), DRD2, DRD3 and DRD4 agonists and antagonists (Fig 8B, 8C, 8D and 8E, respectively). We found that a 2 hr stimulation of macrophages with a DRD1-like agonist SKF 38393 caused a suppression of the CCR5 transcription in control cells, as well as in cells treated with Meth or DA, while the treatment with a DRD1-like antagonist SCH 23390 HCl increased CCR5 transcription compared to untreated conditions (Fig 8B). The treatment of cells with the DRD2 agonist Ropinirole did not affect the expression of CCR5 significantly. At a higher dose it caused a decrease in CCR5 transcription in cells co-exposed to DA. Likewise, the exposure to the DRD2 antagonist Haloperidol did not affect the expression of CCR5 at 2 hrs of treatment in most conditions. At a higher dose, Haloperidol increased CCR5 expression in untreated cells. This suggests that DRD1, and to a lesser degree DRD2, can potentially signal a suppression of CCR5 expression, and may not explain the effect of DA in increasing CCR5 in innate immune cells.

DRD2 stimulation or antagonism did not significantly affect CCR5 expression (Fig 8C). On the other hand, the DRD3 agonist, Promipexole, increased CCR5 expression both in cells that were unstimulated and in cells treated with Meth and DA. The DRD3 antagonist Pnu 177864 decreased CCR5 expression when cells were stimulated with Meth and DA, but not in other conditions (Fig 8D). Regarding DRD4, its agonist PD 168077 robustly increased CCR5 transcription in untreated cells and in cells exposed to Meth or DA, but not in cells exposed to both Meth and DA. In addition, the DRD4 antagonist L 745.870 significantly decreased CCR5 in cells stimulated with DA (Fig 8E). This suggests that signal through DRD3 and especially DRD4 could be involved in the increased transcription of CCR5 that is induced by DA in THP1 cells. Further studies are necessary to determine the pathways involved in these effects.

**Fig 9:**
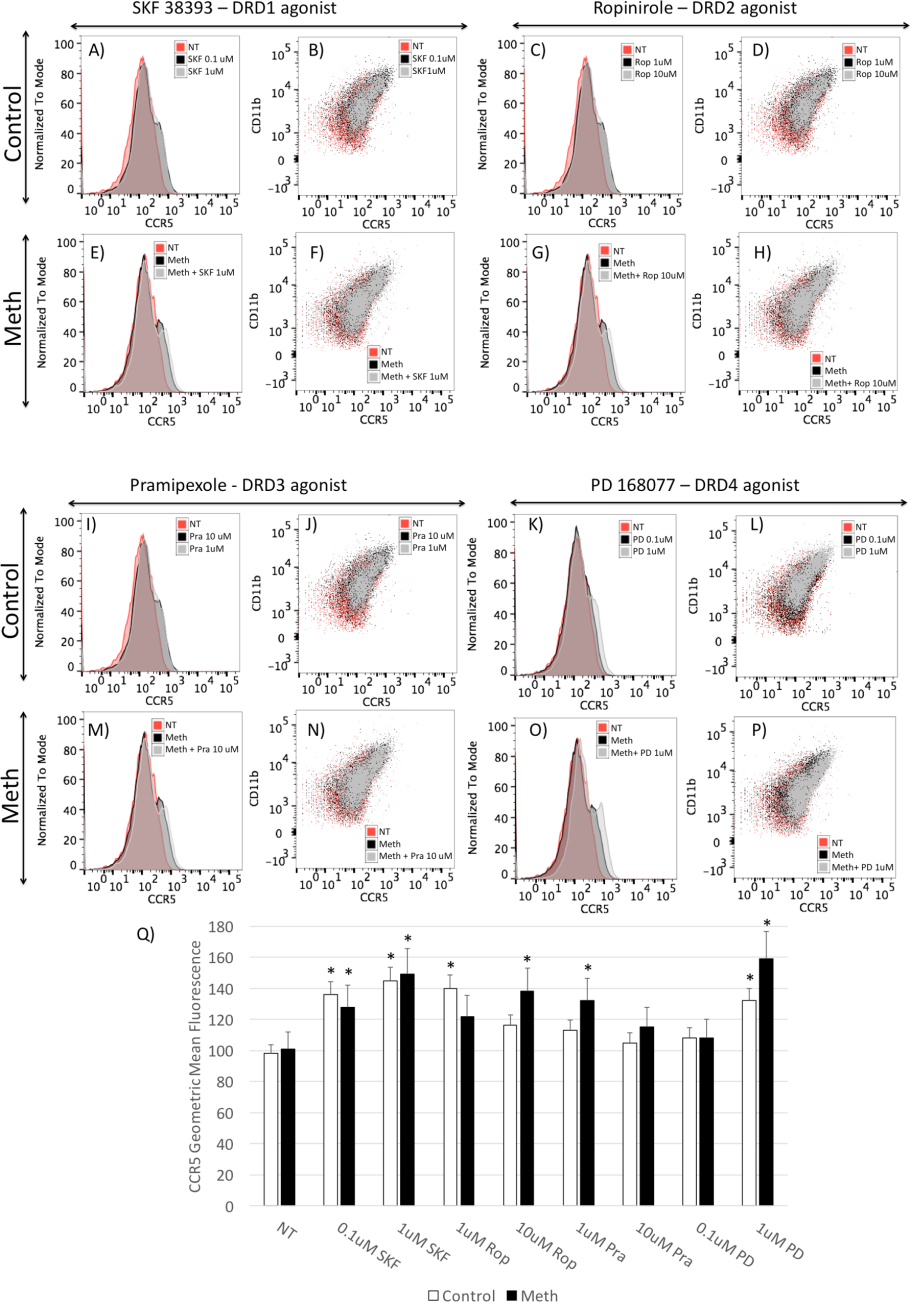
Surface expression of CCR5 in the presence of selective DRD isoform agonists. Control **(A, B, C, D, I, J, K, L)** and Meth-treated **(E, F, G, H, M, N, O, P)** THP1 cells were exposed for 1 hour to DRD1–5 agonist SKF 38393 **(A, B, E, F),** DRD2 agonist **(C, D, G, H),** DRD3 agonist Pramipezole **(I,J, M, N)** and to DRD4 agonist PD 168077 **(K, L, O, P).** The cells were stained with PE- labeled CD11b and APC-labeled CCR5. Scatter plots show the superposition of CCR5 and CD11b subsets in non-treated cells (NT), and cells treated with antagonists in each panel’s legend, with or without simultaneous Meth (60uM). (Q) CCR5 levels measured by the geometric mean fluorescence of cells that received the different DRD agonists, in the presence or absence of Meth. The results represent the average ± SD of 3 independent experiments performed in duplicate. *p<0.05 in Bonferroni’s test, and in comparison to respective controls, or white bars are compared to not treated (NT) without Meth, and black bars are compared to Meth alone NT controls.

In spite of the differential effects of DRD isoform stimulation on the transcription of CCR5, all DA receptor agonists increased the expression of CCR5 at the surface level, as shown by FACS, suggesting translational effects, and in the case of D1-like DRDs, suggesting a dissociation between transcriptional and post-transcriptional effects (Fig 9), or a potential effect on the CCR5 coupling. Interestingly, DRD4 showed the strongest modulation of surface CCR5 expression.

**Fig 10:**
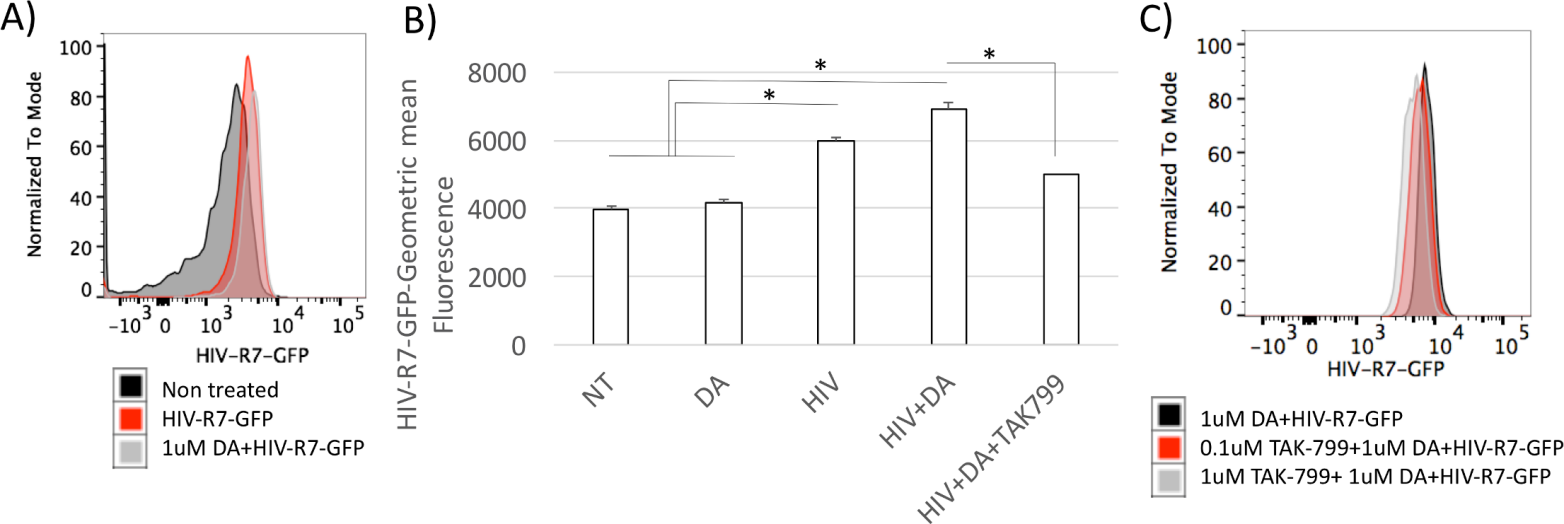
Infection of THP1 cells with HIV is enhanced by DA in a CCR5-dependent fashion. THP1 cells were exposed to HIV-pR7-GFP, and the internalization of viral particles was measured using flow cytometry. (A) Mean fluorescence histogram of non-treated, uninfected cells (black), HIV-infected (red), and HIV-infected in the presence of 1uM of DA. (B) Geometric mean fluorescence of intracellular HIV-pR7-GFP in control cells and cells exposed to DA, in the presence or absence of a CCR5-blocking compound TAK799. Results represent the average and standard deviation of 5 independent experiments performed in duplicate. *p<0.05 in Bonferroni’s post hoc test, where comparisons were assigned to respective controls.

We examined whether the stimulation with DA could increase the susceptibility to HIV infection. For that, we utilized a GFP-tagged HIV construct, HIV-R7-GFP, which can infect innate immune targets and be visualized in infected cells using flow cytometry. Using this system, we observed that the exposure to DA simultaneously to the introduction of virus into the cell culture system, increased the ability of the virus to enter into THP1 cells (Fig 10). The addition of a CCR5 blocker compound, TAK799, prevented the ability of DA to increase intracellular virus, suggesting that the effect of DA is largely dependent on the expression of CCR5.

The expression of CCR5 is important in HIV infection, as it not only promotes inflammation, but also serves as a co-receptor for the viral entry into host cells. In the brain, CCR5+ innate immune cells are the primary targets of HIV, and their number may affect viral load as well as the size of the CNS viral reservoir. Thus, the understanding of mechanisms regulating CCR5 expression is critical. In previous reports, we have found that exposure to Meth, which induces DA in the brain, increases HIV RNA levels locally in correlation with higher gene transcription and surface expression of CCR5 on myeloid cells, microglia and on a subset of intermediately activated macrophages[29].

We have developed a model to study the molecular basis for the transcriptional increase of CCR5, which may ultimately impact the susceptibility to infection in drug abusers. The study was designed to specifically address the upregulation of CCR5 on myeloid cells, which are the predominant targets of HIV in the CNS, so the results cannot be extrapolated to T cells. Using the THP1 human macrophages cell line, we found that Meth and Tat alone do not affect CCR5 gene expression. However, in the presence of DA, acute CCR5 upregulation was robust. This was due to the ability of DA to induce epigenetic modifications throughout the CCR5 promoter, which might facilitate transcription factor binding and RNA Pol accumulation.

The transcription factors affected by Meth and by Tat are relevant to the inflammatory response in innate immune cells, including genes that have a significant impact in HIV infection in the brain, such as CCR5 [30]. Interestingly, the sets of transcription factors activated by Meth differed from the ones activated by DA or by the HIV Tat peptide. We also found that NFkB and GATA3, both activated by Meth directly, were translocated in a ROS-dependent fashion, while CREB and AP1 were activated though a ROS-independent pathway. DA, on the other hand, had a robust dose-dependent effect on cJun, which was stronger than Meth alone, and also was more efficient than Meth at translocating NFkB. HIV Tat increased cJun activation. This suggests that individually, Meth, HIV Tat and DA can affect non-overlapping sets of genes, and together contribute to the cell phenotype.

Importantly, these molecular changes happen following acute exposure, providing insight for the in vivo setting, where the acute induction of neurotransmitters in drug abuse can modulate aspects of the innate immune response, and windows of opportunity for viral entry into host cells, while affecting other cellular functions.

In the CCR5 promoter, epigenetic modifications that enable transcription factor binding were not elicited in the absence of DA, but the induction of important transcription factors was facilitated by Meth regardless of DA. This finding has a profound impact to HIV infection in the context of drugs that induce DA in the brain, and helps explain the increase in viral load, and other aspects of the exacerbated inflammatory neuropathogenesis. These findings are also supported by previous studies in SIV-infected macaques’ brains, where increased CCR5 was correlated with a higher brain viral load [11]. In vivo, CCR5 is increased by Meth regardless of HIV infection, together with pathway components that affect its promoter [12], suggesting the contribution of neurotransmitters such as DA to changes in the innate immune target phenotype.

Together, the relative contribution of DA-dependent and direct Meth stimulation provide an explanation to the Meth-induced increase of CCR5 transcription that is observed in vivo, through its capacity to induce neurons to release post-synaptic DA in critical parts of the brain [31], while increasing NFkB, GATA3, cFos/ cJun and CREB in macrophages and microglial cells [32–34]. Conversely, in this in vitro system, which offers the advantage of a DA-regulated environment and consistency, we found that Meth and/or Tat did not increase RNA polymerase at the CCR5 promoter transcription-starting site, in spite of the transcription factor activation. In the context of DA, though, CCR5 transcription was increased, in correlation with enhancer histone modifications. This suggests that DA, which is increased by drugs such as Meth, is rather critical in the control of the number of HIV target cells, and potentially in the size of the HIV reservoir.

Our findings support the described ability of DA to increase HIV infection in macrophages in a CCR5-dependent fashion [21], but suggest that relative differences in the expression of specific DRD isoforms may be critical to determine the contribution of DA in different systems, as well as to explain individual susceptibilities. We further demonstrate that DA as a whole provides the epigenetic requirements for CCR5 gene transcription in THP1 cells, by promoting enhancer histone modifications that make transcription factor binding sites available within that promoter. Further studies are necessary for understanding the pathways orchestrating the DA-mediated epigenetic control of CCR5, and perhaps of other inflammatory promoters. Together, the results suggest that drugs of abuse such as Meth can potentially affect mechanisms associated with the susceptibility of innate immune cells to HIV infection in a DA-dependent way, by enhancing mechanisms that may increase viral spread such as the expression of the HIV co-receptor CCR5 in brain myeloid cells. These effects of drugs of abuse such as Meth are mediated by a combination of DA neurotransmitter-mediated effects, with consequences to the epigenetic control of the CCR5 promoter and NFkB translocation, and the ability of the drug to directly translocate transcription factors that are relevant within the CCR5 promoter. The stimulation of selective DA receptor (DRD) isoforms increased the density of CCR5 surface expression acutely. However, there was an interesting distinction of the role of individual isoforms in transcription. The results with pharmacological agonists and antagonists suggest that the selective DRD1-5 stimulation may suppress, while DRD4 may increase CCR5 transcription acutely. Thus, the ability of DA to increase or suppress CCR5 could be determined by the relative abundance of these two isoforms, affinity and signaling pathways.

DRD4 has been shown to be a high affinity DA receptor, but in cells where DRD1-5-induced signal prevails, DA could instead provide a suppressing effect. Our results show that in THP1 cells, DRD4 is the most abundant isoform. Interestingly, the treatment of cells with HIV Tat peptide significantly decreased the expression of DRD1 and DRD2, regardless of Meth. This confirms previous in vivo findings, in a transgenic Tat mouse model, where Tat induction in the brain caused a significant decrease in these receptors[35]. The potential implications of this finding are that in the context of HIV infection in the CNS, decreased DRD1 may account for a stronger effect of DA on CCR5+ targets. Nevertheless, all receptors were capable of boosting the surface expression, suggesting translational effects, which remain to be examined. Interestingly, the simultaneous exposure of THP1 cells to HIV Tat and DA, with or without Meth, enhanced the increase in the normalized CCR5 surface density.

Using pharmacological tools we were able to distinguish that DRD1 can downregulate CCR5 transcription, offering an opportunity for therapeutic development. It also suggests that potential changes in the relative abundance or polymorphisms in DRD isoforms [36–38], particularly DRD1, might affect neuropathology and susceptibility to HIV in the brain, as defined by the transcription of CCR5 in myeloid targets. We have previously described that in vivo[35], HIV Tat peptide affects the expression of DRDs. If so, modifications in the abundance of DRD1 expression that can be triggered by HIV and its peptides, and may interfere with a signaling balance between different DRDs, controlling or stimulating pathways that modulate the expression of CCR5 and potentially other inflammatory markers. Thus, the effects of HIV on the DA system could predispose myeloid populations to an increase in the number of target cells, especially upon drug abuse. On the other hand, we have detected a positive effect on CCR5 transcription provided by DRD3 and DRD4. While we have not explored the pathways that are involved in the effects of DRD3 and 4, these receptors have been described in association with other inflammatory markers in human innate immune cells, such as IL6, CCL2, CXCL8 and IL10 [20].

The impact of DA signaling on CCR5 expression was further confirmed using a system to demonstrate the effects on the susceptibility to viral entry, using a GFP-tagged HIV. This confirms and expands previous reports on the participation of DA in viral entry, which was suggested to be through CCR5[21]. The distinction between DRD signaling abilities to promote such effects can be critical for decreasing the burden of HIV in drug abusers.

We do acknowledge that the THP1 system has limitations, and interpretations must be compromised with their immortalized character that makes them monocyte-like cells. Nevertheless, they are commonly used as proxies for the study of human monocytes, although retain very little variation in comparison to human primary cells. On the other hand, in studies where THP1 cells and human primary innate immune cells were compared, primary cells are more responsive to stimuli such as LPS[39]. THP1 cells and human macrophages also differ in their gene expression profiles[40], and are likely different from macrophages that infiltrate the CNS. Yet they provide a consistent system where innate immune signaling and phenotypic behaviors can be thoroughly examined from the point of view of signal transduction and expression phenotypes upon exposure to factors present in the brain environment, and as a first step for understanding the complexity of interactions between drugs of abuse, neurotransmitters and HIV. It is critical to embrace innate immune cells as entities that are responsive to changes in the environment, for instance levels of neurotransmitters, which can modify the course of inflammatory and infectious outcome. Further studies are necessary to understand specific signaling pathways and implications of the DA system to HIV infection of myeloid targets in drug abuse. In addition, other cellular systems need to be examined for determining the value of our findings in the context of active infection, as these cells could not be consistently infected (not shown). In addition, based on the discordance between the ability of DRD1-5 selective agonists to decrease transcription acutely, and yet slightly boost surface expression, the effects of DA and DRD signaling on CCR5 recycling need to be examined.

Altogether, the data suggests in contexts where high levels of DA induction and Meth may co-exist, as in the brain micro-environment of drug abusers, the requirements for acute CCR5 upregulation become fully available, and their regulation is subjected to DRD isoforms expression and signaling efficiency. If this happens in vivo, DA, induced by Meth (and by other drugs of abuse) in areas of the brain with dopaminergic projections, may affect the local availability of HIV targets, with potential consequences to the size of the HIV reservoir, and to the pathogenesis in the CNS [41].

## Supporting information

S1 Fig Image J was used to calculate nuclear translocation of transcription factors, by superimposing a mask designed on the DAPI staining acquisition channel to the transcription factor acquisition channel. The color density was calculated without and with the mask, to determine a ratio between the color intensity in the cytoplasm and in the nucleus, which were integrated to estimate the amount of transcription factor in the nuclear area. A representation of the mask design and estimate of calculations are presented.
(JPG)
