# Supplementary figures and images for: Dopamine and its receptors play a role in the modulation of CCR5 expression in innate immune cells following exposure to Methamphetamine: Implications to HIV infection
Source: PLoS One. 2018 Jun 26;13(6):e0199861. doi: 10.1371/journal.pone.0199861 (PMC6019408; doi:10.1371/journal.pone.0199861)

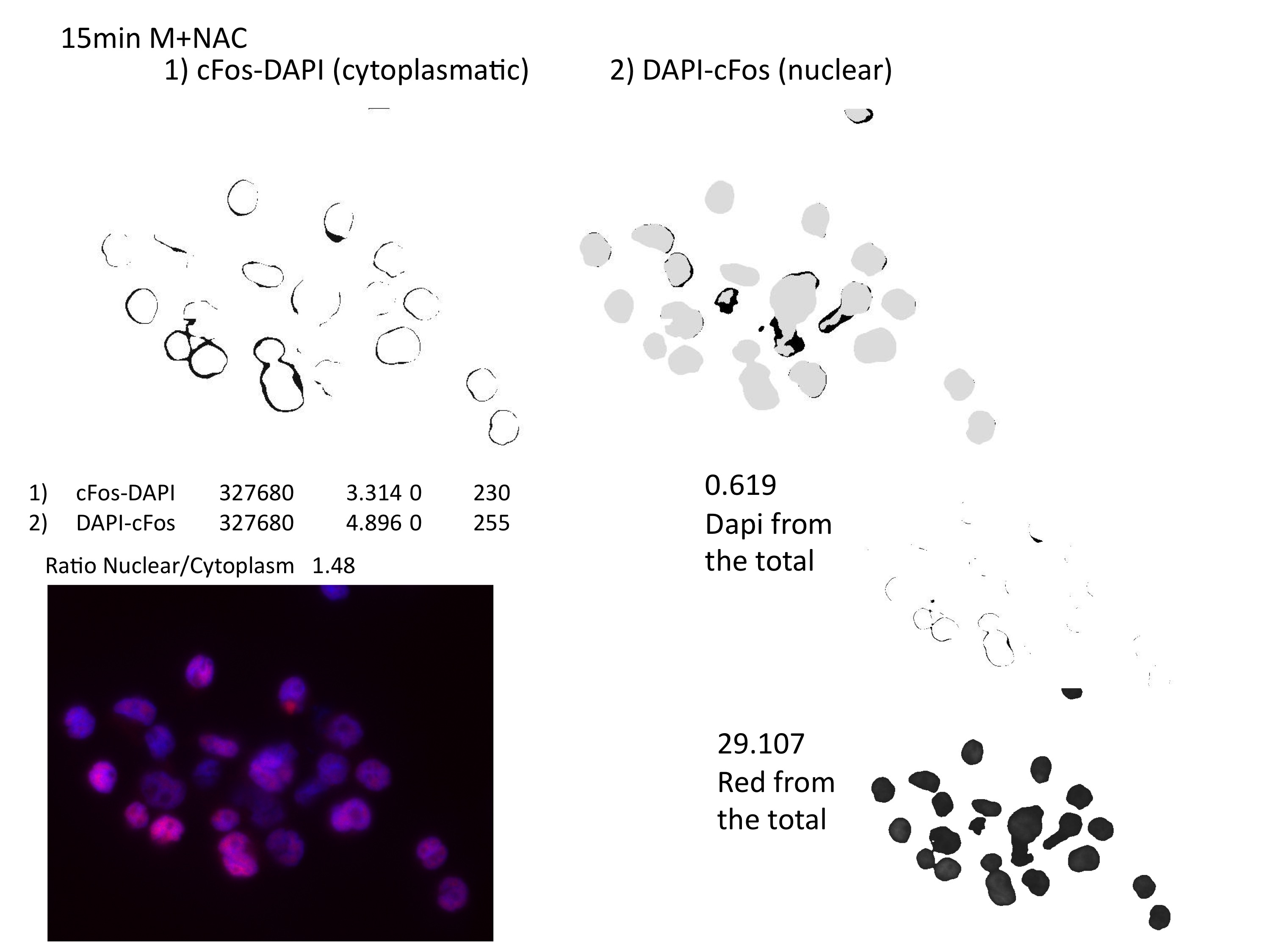

Supplement: S1 Fig — The color density was calculated without and with the mask, to determine a ratio between the color intensity in the cytoplasm and in the nucleus, which were integrated to estimate the amount of transcription factor in the nuclear area. A representation of the mask design and estimate of calculations are presented. (JPG) [file pone.0199861.s001.jpg]
